# Impact of intraoperative hemostatic material placement on intra-abdominal infection control in acute appendicitis: a retrospective cohort study

**DOI:** 10.1186/s12893-026-03886-0

**Published:** 2026-05-30

**Authors:** Haoran Qiao, Xirang Wang, Si Liu, Yuxiang Li, Jian Kang, Xueying Li, Xiaofeng Sun

**Affiliations:** 1 Department of Emergency Medicine, Peking University First Hospital, Beijing, 100034 China; 2 Department of General Surgery, Beijing Fengtai You’anmen Hospital, Beijing, 100069 China; 3 Department of General Surgery, Beijing Huimin Hospital, Beijing, 100054 China; 4 Biostatistics Unit, Peking University First Hospital, Beijing, 100034 China

**Keywords:** Laparoscopic appendectomy, Sodium carboxymethyl cellulose, Absorbable hemostatic gauze, Infectious outcomes, Emergency surgery

## Abstract

**Objective:**

To evaluate the impact of sodium carboxymethyl cellulose (SCMC)-based absorbable hemostatic gauze on postoperative infectious outcomes in emergency laparoscopic appendectomy (LA) for acute appendicitis.

**Methods:**

A retrospective analysis of 184 patients undergoing emergency LA at General Surgery Department of Beijing Fengtai You’anmen Hospital (Collaborative Hospital of Peking University First Hospital Medical Alliance) from January 2024 to March 2025 was conducted. All consecutive patients were included without selection based on disease severity. Patients were divided into two groups: SCMC gauze placement group (*n* = 55) and non-gauze group (*n* = 129). Perioperative parameters were compared between groups using descriptive statistics and simple group comparisons (*t*-test, Mann-Whitney *U* test, *χ²* test).

**Results:**

Baseline characteristics were well balanced between groups (*P* > 0.05 for all). No surgical site infections (SSI) or intra-abdominal abscesses were observed in either group during the three-month follow-up period. Key outcomes showed no statistically significant differences (*P* > 0.05), including: positive rate of intra-abdominal pus culture (97.8% overall); detailed microbial species distribution (e.g., Escherichia coli, Pseudomonas aeruginosa, Klebsiella pneumoniae, Enterococcus spp.); inflammatory markers (WBC, CRP, PCT at 24 h/72 h postoperatively); peak WBC and time to peak; peak body temperature and time to peak; and length of stay (LOS). The distribution of disease severity by AAST CT grade was similar between groups, with no significant difference (*P* = 0.740). All procedures were completed laparoscopically with zero mortality.

**Conclusion:**

The rational use of SCMC-based absorbable hemostatic gauze during emergency LA appears safe, with no observed association between intraoperative gauze placement and increased postoperative infection risk. Given the non-randomized design and zero event rate, these findings should be interpreted as an observational safety signal, not equivalence or antimicrobial benefit.

## Introduction

Laparoscopic appendectomy (LA) has become the standard minimally invasive approach for treating acute appendicitis [1]. However, postoperative infectious complications such as intra-abdominal abscesses and surgical site infections (SSI) remain significant concerns. Achieving effective intraoperative hemostasis is essential to prevent bleeding-related issues. Absorbable hemostatic gauze is an effective hemostatic agent that can substantially reduce bleeding time and blood loss. Studies show it decreases hemostasis time by 33–50% and blood loss by up to 75%. It is especially advantageous in cases of diffuse bleeding or in situations where traditional methods like electrocautery or suturing are challenging, thereby enhancing surgical safety [2–5].

However, concerns remain that hemostatic gauze may exacerbate infections. As a foreign body, hemostatic gauze, due to its surface properties, may promote bacterial colonization and adhesion [6, 7]. Traditional non-antimicrobial gauze especially increases the risk of microbial contamination of wounds [8, 9]. This creates a clinical paradox: the imperative for hemostatic efficacy may compromise antimicrobial integrity, as some materials may create a microenvironment conducive to bacterial growth upon contact with bodily fluids [7, 8]. Therefore, placing gauze inside the abdominal cavity during potentially contaminated surgeries such as appendectomies may further increase the risk of intra-abdominal infection [10, 11].

Critically, the specific impact of intraoperative hemostatic gauze placement on infectious outcomes following emergency LA remains inadequately elucidated. Most existing studies focus on optimizing material performance, while evidence directly correlating gauze placement with infectious sequelae remains limited [11–13]. This study evaluates the influence of sodium carboxymethyl cellulose (SCMC)-based absorbable hemostatic gauze placed during emergency LA on postoperative infectious outcomes, with the goal of providing safety evidence.

## Materials and methods

### Design and ethics

This retrospective cohort study was approved by the Ethics Committees of Peking University First Hospital and Beijing Fengtai You’anmen Hospital (Approval No. 2025-059). The study was conducted in accordance with the Declaration of Helsinki. Written informed consent was obtained from all patients or their legal guardians. The study was supported by the National High Level Hospital Clinical Research Funding (Interdepartmental Research Project of Peking University First Hospital, Grant No. 2024IR10).

### Patients and data collection

We consecutively enrolled all patients who underwent emergency LA for acute appendicitis at Beijing Fengtai You’anmen Hospital between January 2024 and March 2025. No patients were excluded based on culture results. Patients were categorized into two groups according to whether SCMC gauze was used intraoperatively: gauze group (*n* = 55) and non-gauze group (*n* = 129). The decision to place gauze was made by the attending surgeon based on the presence of diffuse microvascular oozing from friable/edematous tissues that did not cease after electrocautery or 30 s of dry gauze compression.

Data were extracted from electronic medical records. Baseline variables included age, sex, body mass index (BMI), comorbidities (hypertension, diabetes, coronary artery disease, etc.), preoperative AAST CT grade, preoperative fever (> 37.3 °C), and preoperative WBC, CRP, and PCT levels. Intraoperative variables comprised operative time, drain placement, irrigation volume, and peritoneal pus culture results. Postoperative variables included WBC, CRP, and PCT at 24 h and 72 h after the operation; peak values and time to peak of WBC and body temperature; length of stay (LOS); and occurrence of SSI or intra-abdominal abscess. All patients underwent routine ultrasonography three months postoperatively to detect asymptomatic fluid collections or abscesses. Postoperative antibiotic duration was also recorded as a descriptive variable.

### Surgical technique and hemostatic gauze details

All procedures were performed by surgical teams with equivalent qualifications. Specifically, all procedures were performed by a single surgical team comprising four attending surgeons (one chief surgeon acting as primary operator and three assistants), all with equivalent qualifications in laparoscopic surgery. The indication for hemostatic gauze placement followed a standardized intraoperative criterion agreed upon before study initiation: persistent microvascular oozing from inflamed or edematous tissue refractory to electrocautery or 30 s of dry gauze compression. A standard three-port technique was employed. The absorbable hemostatic gauze used in this study was Suntouch^®^ (Huizhou Huayang Medical Devices Co., Ltd., China; National Medical Products Administration Registration No. 20143142370). The material is composed of SCMC derived from regenerated cellulose woven fabric via chemical modification. It appears as a white to pale yellow woven sheet. The gauze is sterile, supplied by gamma irradiation, and intended for single use only.

Upon contact with blood or bodily fluids, the gauze rapidly absorbs water, swells, and forms a hydrophilic gel that physically occludes capillary and small vessel bleeding. It gradually dissolves and is absorbed in vivo over 7–14 days. The product has no intrinsic antimicrobial activity, as explicitly stated in the instructions for use. A single piece measuring 10 cm × 7.5 cm was applied intraoperatively in accordance with the manufacturer’s guidance.

Hemostasis was achieved through electrocautery. For diffuse microvascular bleeding in edematous/friable tissues refractory to conventional hemostasis (persistent oozing despite electrocautery or 30 s of dry gauze compression), one piece of hemostatic gauze was placed over the oozing surface. The gauze was firmly apposed without gaps and left in situ. Following hemostasis, the endobag was extracted. Abdominal exudates were cultured for all patients. Systematic irrigation with warm saline was performed until clear effluent was achieved, and a closed-suction drain was placed in the pelvis. The drain was typically removed when output was < 50 mL/24 h and no pus was present (usually on postoperative day 2–3).

### Perioperative antibiotic protocol

All patients received intravenous antibiotics (ceftriaxone 2.0 g) preoperatively within 60 min before incision. Postoperatively, antibiotics were continued for 48 h in uncomplicated cases and for 3 to 5 days in complicated appendicitis (perforation, abscess, or gangrene), tailored to culture and sensitivity results. We acknowledge that routine postoperative antibiotics for uncomplicated appendicitis may not be supported by recent literature; this institutional protocol represents a potential limitation.

### Microbiological assessment

During surgery, peritoneal fluid or pus was aspirated and sent for aerobic and anaerobic culture. Culture positivity rate was recorded; all 184 patients underwent culture. Isolates were identified to species level.

### Observed parameters

The following parameters were recorded and compared: Surgery time; Drain placement rate; Positive rate of intra-abdominal pus culture and detailed microbial species distribution; Inflammatory markers (WBC, CRP, PCT at 24 h/72 h postoperatively); Peak WBC value and time to peak; Peak body temperature and time to peak; LOS; fever duration; postoperative antibiotic duration.

### Statistical analysis

Statistical analysis was performed using SPSS version 23.0. Continuous variables were assessed for normality (Shapiro-Wilk test) and homogeneity of variance (Levene’s test). Normally distributed data with equal variances were analyzed by independent samples *t*-test, expressed as mean ± SD; non-normally distributed data were analyzed by Wilcoxon rank-sum test, reported as median with interquartile range (IQR). For nominal variables, *χ²* test or Fisher’s exact test was selected based on sample size and expected frequencies. For ordinal variables, Mann-Whitney *U* test was employed. The significance level was set at α = 0.05 (two-tailed), with *P* < 0.05 considered statistically significant. No multivariable regression or propensity score weighting was performed, as the absence of clinical infection events rendered such modeling invalid. The analysis focuses on descriptive statistics and simple group comparisons.

## Results

### Baseline characteristics

A total of 184 patients were included; baseline characteristics are summarized in Table 1. All 184 procedures were completed laparoscopically without conversion. There were no significant differences between the gauze and non-gauze groups in age, sex, BMI, comorbidities, preoperative AAST CT grade, postoperative antibiotic duration, febrile status, or preoperative inflammatory markers (*P* > 0.05 for all). Detailed descriptive statistics are presented in Table 1 as n (%) or mean ± SD / median (IQR).

**Table 1:** Baseline Characteristics of Patients Between Groups

	Absorbable Hemostatic Gauze Group (*n* = 55)	No Absorbable Hemostatic Gauze Group (*n* = 129)	Statistic	*P*
Sex, *n* (%)			χ²=1.167	0.280
Male	35(63.6)	71(55.0)		
Female	20(36.4)	58(45.0)		
Age (years)	47.22 ± 15.7	48.18 ± 16.5	*t* = 0.366	0.715
BMI (Kg/m^2^)	25.37 ± 4.27	25.49 ± 4.16	*t* = 0.166	0.868
Comorbidities, n(%)			χ²=2.331	0.127
Yes	28(50.9)	50(38.8)		
No	27(49.1)	79(61.2)		
Preoperative AAST CT Grade, n(%)			*z*=-0.332	0.740
I	17 (30.9)	41(31.8)		
II	12(21.8)	32 (24.8)		
III	20(36.4)	43(33.3)		
IV	4 (7.3)	7(5.4)		
V	2(3.6)	6 (4.7)		
Postoperative antibiotic duration (days)			*z*=-1.876	0.061
1	2(3.6)	9(7.0)		
2	5(9.1)	16(12.4)		
3	17(30.9)	50(38.7)		
4	17(30.9)	31(24.0)		
5	7(12.7)	15(11.6)		
6	3(5.5)	3(2.3)		
7	3(5.5)	2(1.6)		
8	1(1.8)	1(0.8)		
10	0(0.0)	2(1.6)		
Preoperative Temperature (°C), n(%)			χ²=2.100	0.147
> 37.3	16 (29.1)	25 (19.4)		
≤ 37.3	39(70.9)	104(80.6)		
Peak Preoperative Temperature (°C)	36.80(36.70,38.00)	36.90(36.80,37.50)	*z*=-1.526	0.127
Preoperative WBC(×10^9^/L)	12.42 ± 4.17	11.86 ± 3.88	*t*=-0.874	0.383
Preoperative CRP(mg/L)	133.49(78.52,222.51)	131.04(92.85,189.99)	*z*=-0.009	0.993
Preoperative PCT (ng/ml)	2.05(1.85,2.27)	2.03(1.88,2.17)	*z*=-0.477	0.634

### Intraoperative and postoperative outcomes

Table 2 displays the comparison of intraoperative and postoperative parameters. Operative time, drain placement rate, irrigation volume did not differ significantly between groups. No clinical SSI or intra-abdominal abscess was observed in either group during the three-month follow-up period. Postoperative inflammatory markers at 24 h and 72 h, peak WBC, time to peak WBC, peak body temperature, time to peak temperature, and LOS were comparable between groups (*P* > 0.05 for all).

**Table 2:** Comparison of intraoperative and postoperative parameters between groups

	Absorbable Hemostatic Gauze Group (*n* = 55)	No Absorbable Hemostatic Gauze Group (*n* = 129)	Statistic	*P*
Surgery time (hours)	1.32(1.08,1.50)	1.20(1.03,1.47)	z=-1.381	0.167
Drain, n(%)			χ²=0.553	0.457
Placed	38(69.1)	96 (74.4)		
Not Placed	17(30.9)	33 (25.6)		
WBC at 24 h Postoperative (×10^9^/L)	11.43 ± 3.86	10.96 ± 3.74	*t*=-0.760	0.448
CRP at 24 h Postoperative (mg/L)	78.24(28.21,177.48)	77.09(35.19,151.08)	*z*=-0.404	0.686
PCT at 24 h Postoperative (ng/ml)	1.54(1.23,1.74)	1.52(1.25,1.66)	*z*=-0.493	0.622
WBC at 72 h Postoperative (×10^9^/L)	8.50 ± 1.72	7.97 ± 2.18	*t*=-1.584	0.115
CRP at 72 h Postoperative (mg/L)	43.89(20.58,53.82)	42.07(25.16,51.69)	*z*=-0.617	0.537
PCT at 72 h Postoperative (ng/ml)	0.86(0.72,0.94)	0.86(0.73,0.90)	*z*=-0.537	0.591
Peak Postoperative WBC (×10^9^/L)	11.63 ± 3.87	11.59 ± 3.60	*t*=-0.058	0.954
Timing of Peak WBC (POD), n(%)			*z*=-0.182	0.856
POD 0	12(21.8)	22(17.1)		
POD 1	36(65.5)	97(75.2)		
POD 2	1(1.8)	3(2.3)		
POD 3	2(3.6)	0(0.0)		
POD 4	1 (1.8)	0(0.0)		
POD 5	0(0.0)	0(0.0)		
POD 6	0(0.0)	0(0.0)		
POD 7	0(0.0)	1(0.8)		
Peak Postoperative Temperature (°C)	36.70(36.60,38.00)	36.70(36.60,37.00)	*z*=-0.503	0.615
Timing of Peak Temperature (POD), n(%)			*z*=-0.565	0.572
POD 0	33(60.0)	71(55.0)		
POD 1	14 (25.5)	37(28.7)		
POD 2	4(7.3)	12(9.3)		
POD 3	2(3.6)	6 (4.7)		
POD 4	2(3.6)	2(1.6)		
POD 5	0(0.0)	0(0.0)		
POD 6	0(0.0)	1(0.8)		
LOS (days)	4.00(3.00,5.00)	4.00(3.00,4.50)	*z*=-1.646	0.100

Intraoperative cultures (presented in Table 3) were obtained before gauze placement and reflect pre-existing contamination. They serve only to demonstrate baseline comparability and do not assess the effect of the hemostatic material on postoperative infection.

**Table 3:** Intraoperative microbial culture results

	Absorbable Hemostatic Gauze Group (*n* = 55)	No Absorbable Hemostatic Gauze Group (*n* = 129)	Statistic	*P*
Microbial Classification, *n*(%)			-	0.115
Gram-negative Bacilli				
Escherichia coli	30(54.6)	90(69.5)		
Pseudomonas aeruginosa	10(18.2)	10(7.8)		
Klebsiella pneumoniae	3(5.5)	5(3.9)		
Citrobacter spp.	1(1.8)	6(4.7)		
Comamonas testosteroni	2(3.6)	3(2.3)		
Klebsiella oxytoca	2(3.6)	2(1.6)		
Proteus mirabilis	3(5.5)	1(0.8)		
Other Gram‑negative bacilli	2(3.6)	4(3.1)		
Gram-positive Cocci				
Enterococcus avium	1(1.8)	2(1.6)		
Enterococcus faecium	1(1.8)	1(0.8)		
Other Gram‑positive cocci	0(0.0)	5(3.9)		

## Discussion

This study evaluated the influence of intraoperative SCMC-based absorbable hemostatic gauze (Suntouch^®^) on postoperative infectious outcomes following emergency LA. In a cohort of 184 patients, we observed no significant differences in baseline characteristics or in a wide range of infection-related parameters between groups. Although no inferential modeling was conducted, neither group experienced clinical surgical site infections or abdominal abscesses, which provided a safety signal for the use of hemostatic gauze.

Sodium carboxymethyl cellulose (SCMC), as an anionic polysaccharide derivative, exerts its hemostatic efficacy through multiple mechanisms: its high hydrophilicity promotes blood concentration and rapid aggregation of erythrocytes and platelets (with adsorption rates of 93% and 80%, respectively) [14]; the carboxyl groups synergize with calcium ions to activate the coagulation cascade, significantly shortening clotting time to approximately 20 s [15]; and its porous sponge-like structure efficiently absorbs exudate, providing a physical barrier over the wound surface [16]. Of note, in contaminated surgical settings, hemostatic materials lacking antimicrobial functionality may become a nidus for infection. Although the SCMC gauze used in this study is anionic, its structural optimization (e.g., high porosity, rapid fluid absorption) reduces blood stasis and indirectly inhibits bacterial colonization. In contrast, cationic materials (e.g., chitosan derivatives) can actively disrupt bacterial membrane integrity through electrostatic interactions, thereby exerting intrinsic antimicrobial activity [17, 18], while oxidized regenerated cellulose (ORC) achieves broad-spectrum bacteriostatic effects via its acidic microenvironment and oxidative properties. Future research may explore compositing SCMC with trace amounts of cationic polymers (e.g., quaternized chitosan) to further enhance its anti-infective capacity in contaminated wounds [19, 20]. The manufacturer’s instructions do not claim safety in infected environments, and no antimicrobial activity is asserted. Our study therefore provides an independent observational safety signal rather than manufacturer-endorsed evidence.

LA for acute appendicitis is classified as a clean-contaminated procedure, wherein intraoperative peritoneal exposure and tissue manipulation substantially increase the risk of SSI [21]. Traditional non-absorbable materials (e.g., conventional bone wax) may induce foreign-body reactions, impede tissue healing, and serve as a scaffold for bacterial biofilm formation owing to their biological inertness and persistence [22]. Multiple studies have confirmed that retained material is an important cause of postoperative imaging misinterpretation (e.g., mistaking residual hemostatic agent for an abscess) and chronic inflammation [23]. The SCMC gauze employed in this study is fully absorbable, thereby eliminating the infectious risk associated with long-term foreign-body retention. Animal experiments have shown that modified cellulose materials such as partially carboxymethylated cellulose degrade safely at the wound site without significant tissue toxicity [24], which aligns with the absence of material-related complications observed in our series. Moreover, the rapid swelling property of SCMC helps seal microvessels on the wound surface and reduces the opportunity for bacterial dissemination within the operative field [25, 26].

In emergency abdominal surgery, hemostatic agents must balance effective hemostasis, biocompatibility, and anti-infective properties. ORC, a classic absorbable hemostat, has been shown to reduce postoperative adhesions and infection risk, but its acidic degradation products may cause local irritation. Chitosan-based materials exhibit strong tissue adhesion and cationic antimicrobial activity, performing well in high-bleeding-risk models such as liver injury [17]; however, their high cost and stability in the strongly acidic peritoneal environment (e.g., perforated appendicitis) require further validation. SCMC gauze achieved rapid hemostasis during appendiceal stump management (mean hemostasis time < 30 s) without increasing postoperative complications (SSI rate 1.2% vs. 1.3% in the control group) [27], consistent with safety data reported for polymeric clips and endoscopic staplers in laparoscopic appendectomy studies [28]. Notably, SCMC barrier membranes (e.g., hyaluronate-CMC composite films) have been proven to significantly reduce the risk of early postoperative bowel obstruction in abdominopelvic surgery [29], suggesting potential for adhesion prevention that warrants further investigation in appendectomy settings.

Foreign-body retention constitutes a fundamental pathological basis for SSI. Experimental studies indicate that surface roughness, hydrophilicity/hydrophobicity, and charge characteristics directly influence bacterial adhesion: anionic materials such as SCMC reduce non-specific protein adsorption and thereby decrease initial adherence of Staphylococcus aureus and Escherichia coli [30, 31]; conversely, CMC films incorporating nano-silver or graphene oxide further inhibit biofilm formation through sustained release of antimicrobial ions [30]. In animal infection models, CMC/alginate films containing ε-polylysine have demonstrated potent inhibitory effects against common abdominal pathogens (e.g., E. coli), providing a rationale for developing infection-resistant hemostatic gauzes. The rapid absorption profile of the SCMC gauze used here (typically degrading within 7–14 days) markedly shortens the duration of foreign-material exposure, thereby reducing infection risk from the source, aligning with the established principle that briefer exposure reduces the likelihood of infection [32, 33].

As noted, SCMC gauze was used selectively in cases with diffuse oozing, introducing inherent indication bias. A key limitation is the absence of an objective intraoperative bleeding severity score or estimated blood loss measurement. Overall blood loss was minimal in all cases, but precise quantification was not routinely recorded in electronic medical records, and no validated bleeding severity score was used intraoperatively. Without such quantification, residual confounding by unmeasured factors (e.g., bleeding severity, surgeon experience, tissue friability) remains possible.

The gauze group comprised only 55 patients, which limits the ability to detect small differences in infection-related outcomes. Given the zero event rate for clinical infections, formal power analysis is not applicable, but it is evident that the study is underpowered for rare complications such as intra-abdominal abscess (expected incidence 3–5%). Therefore, the absence of observed infections does not imply equivalence, and a type II error is possible. Larger prospective studies are needed to confirm these findings.

Our conclusions are specific to the SCMC-based gauze and should not be generalized to other hemostatic agents with different compositions (e.g., ORC, chitosan-based, or antimicrobial-impregnated materials). The product’s lack of antimicrobial activity distinguishes it from materials that actively inhibit bacterial growth.

Beyond those discussed, this study has several limitations: retrospective single-center design, potential residual confounding, short follow-up period (may miss very late abscesses).

This study provides an observational safety signal that SCMC-based absorbable hemostatic gauze, when used judiciously in emergency LA, does not adversely affect postoperative infectious outcomes. Given the non-randomized design and zero event rate, the findings should be interpreted as product-specific safety evidence rather than proof of equivalence or antimicrobial benefit. Future prospective studies with larger samples and longer follow-up are warranted to compare different hemostatic agents in contaminated fields.

## Data Availability

All data generated during this study are included in this published article.

## Abbreviations

LA: Laparoscopic appendectomy
WBC: White blood cell
CRP: C-reactive protein
PCT: Procalcitonin
LOS: Length of stay
SSI: Surgical site infections
BMI: Body mass index
AAST: American association for the surgery of trauma
CT: Computed tomography
SD: Standard deviation
IQR: Interquartile range
SCMC: Sodium carboxymethyl cellulose
ORC: Oxidized regenerated cellulose
POD: Postoperative day
